# Terrestrial actinomycetes from diverse locations of Uttarakhnad, India: Isolation and screening for their antibacterial activity

**Published:** 2013-09

**Authors:** Vijay Kumar, Gajraj Singh Bisht, Omprakash Gusain

**Affiliations:** 1 Department of Microbiology, Sardar Bhagwan Singh Post Graduate Institute of Biomedical Sciences and Research, Balawala, Dehradun, Uttarakhand, India, 248161; 2 Department of Zoology and Biotechnology, H.N.B. Garhwal University, Srinagar, Uttarakhand, India

**Keywords:** Actinomycetes, Altitudinal variation, Antibacterial activity, *Streptomyces*

## Abstract

**Background and Objective:**

Uttarakhand region is less explored, but possess a great biodiversity. This diversity can be explored for isolation and characterization of new actinomycetes strains for seeking antimicrobial molecules. It can therefore be predicted that novel bioactive metabolite producing actinomycetes can be discovered to combat multidrug resistant bacterial pathogens.

**Materials and Methods:**

Variations in the viable count of actinomycetes were accessed in different altitudes. Actinomycetes were isolated, indentified and screened for their antibacterial activity.

**Results:**

The highest viable counts of actinomycetes were recorded in valleys followed by mid hills and high hills. A total of 512 actinomycetes were isolated which were found to belong the 14 different genera of actinomycetes. Mainly the genus *Streptomyces* was dominant in all the soil samples. Out of 512 isolates recovered, 23.44% exhibited antibacterial activity against one or more tested bacterial pathogens. Of these 56.67% showed activity against Gram-positive bacteria, 26.67% against Gram-negative bacteria while 16.67% showed broad spectrum activity. Isolate DV1S and GR9a-5 showed highest antibacterial properties against several multi-drug resistant bacterial pathogens and were identified using polyphasic approach. DV1S and GR9a-5 were found to be most closely related with *S. massasporeus* NBRC 12796^T^ and *Nocardia nova* JCM 6044^T^ respectively.

**Conclusion:**

The results of this study strongly support the idea that the viable count of actinomycetes varied greatly with altitude. The actinomycetes species isolated from valleys, mid hills and high hills possess significant capacity to produce compounds which are active against several drug resistant bacterial pathogens.

## INTRODUCTION

The emergence of multidrug resistant among common bacterial pathogens is a serious problem. Therefore, there is a continuous need for new molecules to combat these pathogens. This need for new antibiotics for the past few decades has been met largely by semisynthetic tailoring of natural product scaffolds discovered in the middle of the 20^th^ century (1). As the soil-derived microorganisms have been intensively screened as a source of therapeutically important molecules over a half century (2), the frequency of discovering structurally new compounds is decreasing these years. These findings seem to imply that the easily accessible microorganisms in soil had been exhausted and there is a need to seek unutilized microorganisms from unexplored sources. It is likely that the diversity of secondary metabolites relies more or less on the isolation source, namely, the habitat of the producers (3). On the basis of above facts new actinomycetes strains that generate active compounds have been recently isolated from novel sources including saline, ocean, mangrove forests and niche habitats such as caves, beehives, solitary wasp mud nest, earthworm castings, pristine forests, lakes, rivers and other wetlands (4–9). New species of the microorganisms have the potential to produce new metabolites, which justifies the isolation of new species for drug discovery purposes (10). In addition, the isolation of diverse strains of actinomycetes provides information for exploitation and utilization of resources produced by this group of microorganisms (11).

To cope up with the demand for new pharmaceutical compounds and to combat the antibiotic resistant pathogens, researchers have been forced to look for novel microorganisms in unexplored environments. Therefore, the soil samples from different regions of the Himalayan state of Uttarakhand was collected for isolation and screening. Uttarakhand region though less explored, but possess a great biodiversity. This diversity can be explored for isolation and characterization of native actinomycetes for antibacterial molecules. Nevertheless, detailed studies on occurrence and distribution of actinobacteria in valleys, mid hills, and high hills of Uttarakhand are still lacking. As per our knowledge we are first time reporting the culturable actinobacterial diversity and their antibacterial activities from these sites.

## MATERIALS AND METHODS

### Collection of soil samples

Soil samples were randomly collected from different locations of Uttarakhand, India. The sampling sites are given in Table 1. Three samples were collected from each site and were carefully taken with spatula after removing 2-3 mm top soil and kept in sterile polypropylene bags. The collected samples were taken to the laboratory for isolation of actinomycetes. Totally, 60 soil samples (3 from each site) were collected from different areas / locations of Uttarakhand.

**Table 1:** Details of soil samples collected from different locations of Uttarakhand.

	Soil sample collection sites	pH*	Altitude(Meters)
Valleys(below 700m)	Rishikesh	7.9±1.0	352
	Dehradun	7.8±2.0	623
	Srinagar	8.2±1.0	577
	Devprayag	7.5±0.5	484
Mid Hills(800-2000m)	Bageshwar	6.6±2.0	890
	Narendranagar	8.0±1.0	1070
	Almora	7.5±1.0	1457
	Uttarakashi	6.9±2.0	1473
	Chamba	7.7±1.0	1583
	Pauri	7.6±1.0	1619
	Tehri	7.7±1.0	1631
	Ranikhet	7.3±0.5	1779
High Hills(above 2000m)	Munsyari	7.9±1.0	2093
	Gangnani	7.6±1.0	2108
	Jhala	8.6±1.0	2455
	Dharali	7.4±0.5	2534
	Lanka	6.0±1.0	2618
	Gangotri	6.0±1.0	2982
	Badrinath	7.8±2.0	3090
	Mana	7.6±2.0	3212

* Mean ± Standard deviation

### Isolation and screening of actinomycetes for their antibacterial activity

Isolation of actinomycetes was done according to the method described previously (9). The preliminary antibacterial activity was checked by agar disc method (12). The bacterial cultures used in the study *Staphylococcus aureus* MTCC96, *Micrococcus luteus* MTCC106, *Bacillus subtilis* MTCC441, *Escherichia coli* MTCC2939, *Pseudomonas aeruginosa* MTCC424, *Acinetobacter baumanii* MTCC1425 (resistant to cephotaxime and streptomycin) and *Mycobacterium smegmatis* MTCC6 were procured from Microbial Type Culture Collection (MTCC), Chandigarh, India while clinical isolates of *S. aureus* (methicillin resistant *S. aureus*), *E. coli* (resistant to co-trimoxazole, bacitracin, erythromycin, cephalothin and penicillin-G) and *Acinetobacter* sp. (resistant to cephotaxime and nitrofuratoin) from Departmental culture collection, Department of Microbiology, Sardar Bhagwan Singh PG institute of Biomedical Sciences and Research, Balawala, Dehradun, India.

### Extraction of metabolites from solid agar media

The isolates showing activities in plug were selected for extraction with n-hexane, ethyl acetate and methanol according to method described previously (8). The Petriplates left after cutting the agar plugs were flooded with n-hexane, ethyl acetate and methanol separately in each plate and left at room temperature for an hour, then crushed with glass rod and filtered with Whatman no.1. The solvents were evaporated under vacuum and dried.

### Antibacterial activity of extracted product

The anti-bacterial disk diffusion assay was carried out on Mueller–Hinton agar (HiMedia) plates following the method described previously (13). The stock (25 mg/ml in DMSO) of actinomycete extract was prepared. The 10 µl of extract was impregnated on sterile discs (6 mm diameter, Whatman paper) and allowed to dry for 30 min. The discs were transferred to the surface of bacterial lawn. The disks containing solvent (DMSO) served as negative control. The disk containing antibiotic rifampicin (5 µg/disc, HiMedia) and vancomycin (30 µg/disc, HiMedia, India) were used as positive controls. The plates were then incubated for 24 h at 37°C, and the zone of bacterial growth inhibition around disk was measured. The assay was repeated twice, and mean of the three experiments was recorded.

### Identification of Actinomycetes

All strains were characterized morphologically and physiologically according to the methods described in the International *Streptomyces* project (14) and Bergey's Manual of Systematic Bacteriology (15). The spore chain morphology and spore surface ornamentation was examined by scanning electron microscopy according to the method described previously (16). The cell wall diamiopimelic acid isomers, whole cell sugars analysis and 16S rDNA sequence analysis was done as described in previous study (8).

### Statistical Analysis

The one-way analysis of variance (ANOVA) was carried out on the viable count of actinomycetes (CFU g^-1^) to see the significant difference among three altitudes (valleys, mid hills and high hills). Further Post Hoc analysis was done to see the significance difference between altitudes (valleys, mid hills and high hills). The software used for computation of statistics was SPSS version 16.

## RESULTS

Uttarakhand hills (including foot hills) though less explored but possesses a great biodiversity had been selected for the present study. A total of 60 soil samples were randomly collected from 20 different locations of Uttarakhand, India. The pH of soil samples ranged between 6.5 and 8.6. When considering the pH of the soil samples relatively highest viable count of actinomycetes were recovered at alkaline (Above 7.5) while lowest from acidic soil. Highest viable count of actinomycetes were recorded in valleys (1.19 ×10^5^±34.94) followed by mid hills (4.6×10^4^±15.02) and low hills (3.6×10^4^±11.36) (Table 2). The significant difference in the viable count of actinomycetes were recorded for three different altitude (F (2,177) = 231.76, p < 0.000). On the basis of different macromorphology, a total of 512 actinomycetes were isolated. These isolates were found to belong to the 14 different genera of actinomycetes. Out of 512 isolates, 65.23% (n = 334) were *Streptomyces*, 10.35% (n = 53) *Streptosporangium*, 8.98% (n = 46) *Actinomadura*, 4.49% (n = 23) *Nocardia*, 2.73% (n = 14) *Nocardiodes*, 1.95% (n = 10) *Saccharopolyspora*, 1.36% (n = 7) *Thermoactinomycetes*, 1.17% (n = 6) *Amycolatopsis*, 0.97% (n = 5) *Micromonospora*, 0.97% (n = 5) *Microbispora*, 0.78% (n = 4) *Intrasporangium*, 0.58% (n = 3) *Planobispora*, 0.195% (n = 1) *Nocardiopsis* and 0.195% (n = 1) *Geodermatophilus* (Table 3).

**Table 2:** Variations in viable count of actinomycetes with altitudes.

Sites	Plate count (CFU g^-1^)× 10^3^	No. of isolates	Active isolates
Valleys(below 700 m)	119.3±34.94*	203	29.55%
Mid hills(800-2000 m)	46.48±15.02*	190	21.05%
High Hills(above 2000)	36.52±11.36*	119	16.88%
	Total	512	23.43%

Mean ± standard deviation

* Viable counts of actinomycetes are significantly different in three different sites (one way ANOVA, Tukey HSD, P < 0.05).

**Table 3:** Distribution of genera of actinomycetes from soil samples collected from different regions of Uttarakhand.

Sampling area	No. of isolates	Number of actinomycetes isolated													
		A	B	C	D	E	F	G	H	I	J	K	L	M	N
Rishikesh	55	34	11	2	1	1	3	-	-	1	1	-	-	-	1
Dehradun	69	30	9	10	7	3	2	4	2	-	-	2	-	-	-
Shrinagar	49	31	9	5	-	3	-	1	-	-	-	-	-	-	-
Deoprayag	30	14	3	7	2	-	1	1	-	-	1	-	-	1	-
Bageshwar	10	8	-	2	-	-	-	-	-	-	-	-	-	-	-
Narendernagar	20	18	-	-	1	-	-	-	-	-	-	-	1	-	-
Almora	21	16	1	2	2	-	-	-	-	-	-	-	-	-	-
Uttarkashi	18	12	-	2	2	-	-	-	-	1	1	-	-	-	-
Chamba	27	25	-	1	1	-	-	-	-	-	-	-	-	-	-
Pauri	39	20	9	5	2	3	-	-	-	-	-	-	-	-	-
Tehri	35	21	2	5	1	1	1	-	2	1	1	-	-	-	-
Ranikhet	20	15		2	-	1	-	-	-	-	-	-	-	-	-
Munsyari	20	18	2	-	-	1	1	-	-	-	-	-	-	-	-
Gagnani	9	5	1	1	-	1	-	-	-	-	-	1	-	-	-
Jhala	6	4	-	-	1	-	-	-	-	-	-	-	1	-	-
Dharali	23	15	1	-	-	-	-	-	2	2	1	1	1	-	-
Lanka	10	9	-	-	-	-	1	-	-	-	-	-	-	-	-
Gangotri	13	12	-	-	1	-	-	-	-	-	-	-	-	-	-
Badrinath	21	17	1	2	1	-	-	-	-	-	-	-	-	-	-
Mana	17	10	4	-	1	-	1	1	-	-	-	-	-	-	-
Total	512	334	53	46	23	14	10	7	6	5	5	4	3	1	1

-, No isolates were recovered; A, *Streptomyces*; B, *Streptosporangium*; C, *Actinomadura*; D, *Nocardia*; E, *Nocardiodes*; F, *Saccharopolyspora*; G, *Thermoactinomycetes*; H, *Amycolatopsis*; I; *Micromonospora*; J, *Microbispora*; K, *Intrasporangium*; L, *Planobispora*; M, *Nocardiopsis*; N, *Geodermatophilus*

**Fig. 1:**
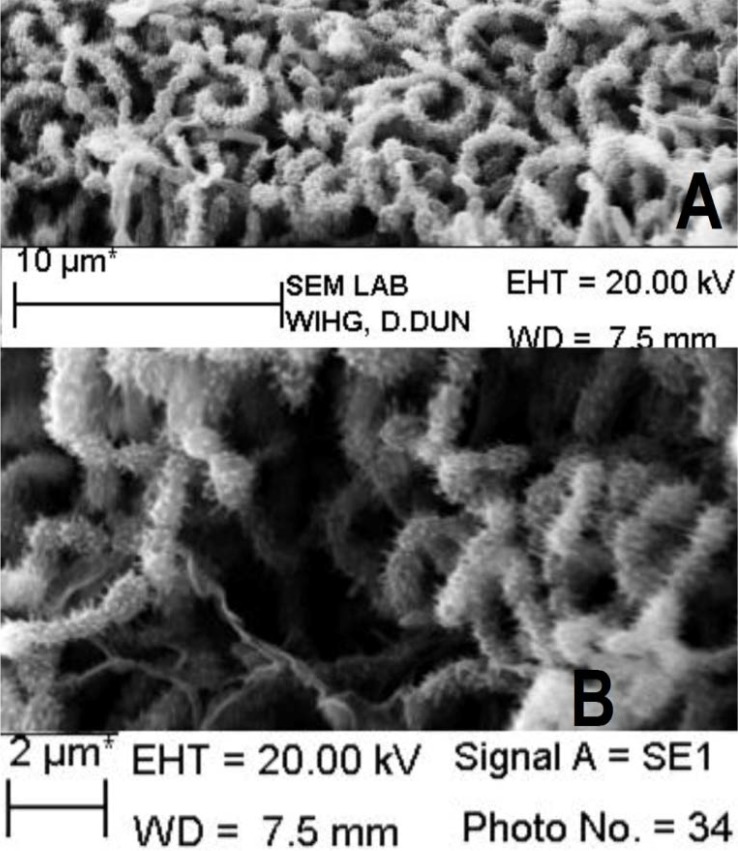
Scanning electron micrograph showing spore chain morphology (A) and spore surface ornamentation (B) of *Streptomyces* sp. DV-1S

**Fig. 2:**
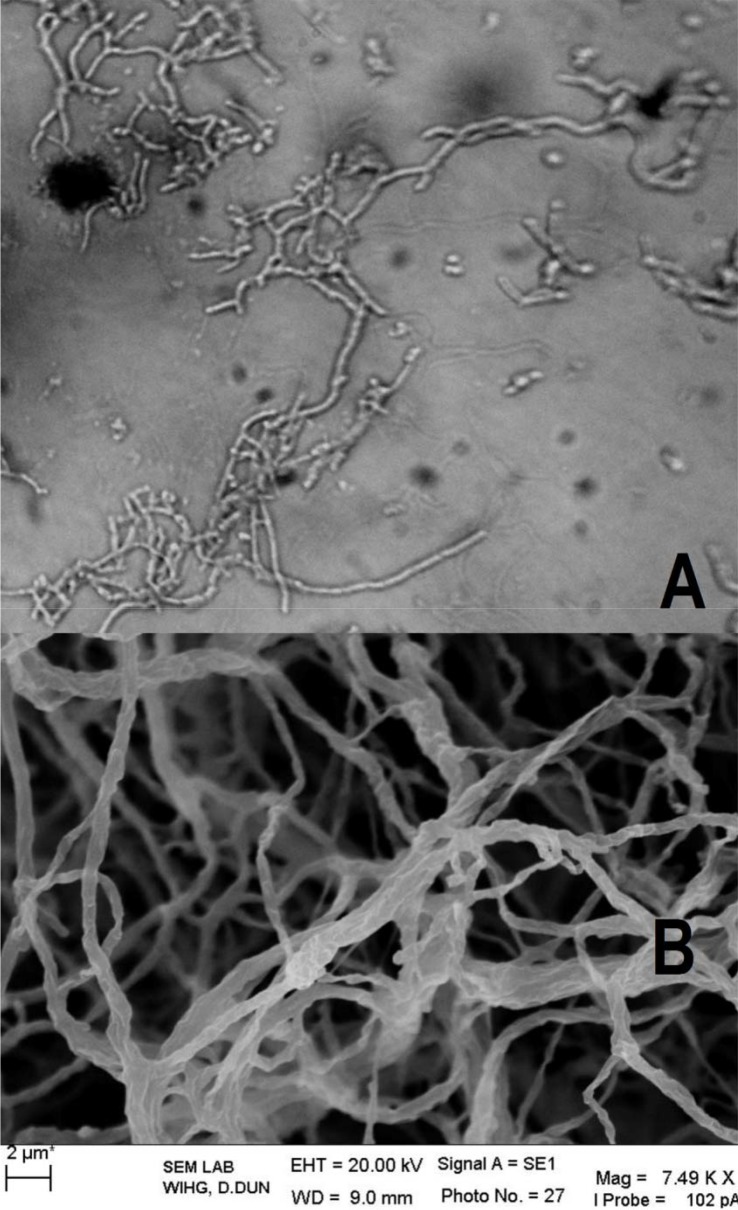
A: Microphotograph showing fragmentation of aerial mycelium; B: scanning electron micrograph showing non-sporulating aerial mycelium of *Nocardia* sp. GR9a-5, Bar, 2 µm

**Fig. 3:**
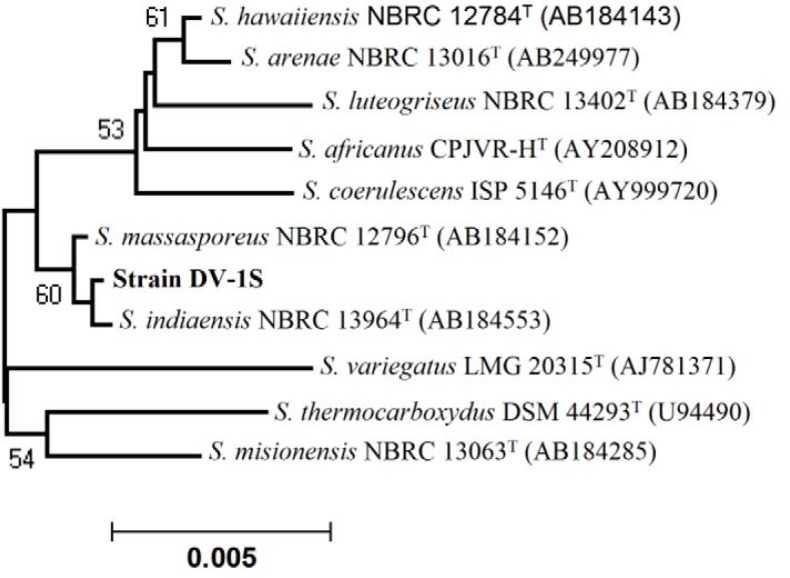
Neighbour-joining phylogenetic tree based on 16S rDNA gene sequences showing the relationships between strains DV-1S and the most closely related type strains of *Streptomyces*.

**Fig. 4:**
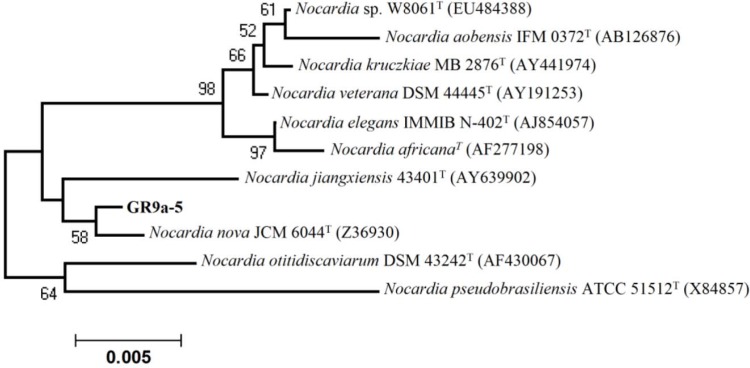
Neighbour-joining phylogenetic tree based on 16S rDNA gene sequences showing the relationships between strains GR9a-5 and the most closely related type strains of *Nocardia*.

Out of 512 isolate, 23.44% (n = 120) exhibited antibacterial activity against one or more tested bacterial pathogens. Of these, 56.67% (n = 68) showed activity against Gram-positive bacteria (*S. aureus, B. subtilis, M. luteus*), 26.67% (n = 32) against Gram-negative bacteria (*E. coli, P. aeruginosa, A. junii*) while 16.67% (n = 20) showed broad spectrum (both Gram-positive and Gram-negative) activity. Some of the active isolates are depicted in Table 4. Isolates DV1S and GR9a-5 were found to be most prominent in the terms of desirable activities, hence taken for further studies. These isolates were found to have promising antibacterial properties against several drug resistant bacterial pathogens (Table 4) and were identified using polyphasic approach. The aerial mycelium of DV1S have open spiral with spiny spores (Fig. 1) while the isolate GR9a-5 lack aerial mycelium with spores (Fig. 2). The physiological characteristics of strain DV1S and GR9a-5 are given in Table 5. Chemotaxonomic tests showed that whole-cell hydrolysates of isolate DV1S were rich in LL- diaminopimelic acid (LL-DAP), while no characteristic sugar indicated a chemotype I. The whole-cell hydrolysates of isolate GR9a-5 were rich in meso- diaminopimelic acid (meso-DAP), along with arabinose and galactose indicated a chemotype IV. On the basis of chemotaxonomic, morphological, and physiological properties of the isolate DV-1S and GR9a-5 are in line with its classification in the genus *Streptomyces* and *Nocardia* respectively. The 16S rDNA sequences (900 bp) of the strains DV1S and GR9a-5 were determined and submitted to GenBank under the accession number HM991289, HM991288. Isolate DV1S shares a sequence similarity of 99.6% with *S. massasporeus*. Its position among the type strains of *Streptomyces* is shown in Fig. 3. While the isolate GR9a-5 shared 16S rRNA gene sequence similarity of 99.33% with *Nocardia nova* JCM 6044^T^. Its position among the type strains of *Nocardia* is shown in Fig. 4.

**Table 4:** Antibacterial activity of some promising isolates

Isolates		Test microorganisms ( Inhibition zone diameter in mm)								
	*S. aureus* MTCC 96	*S. aureus* (MRSA)	*B. subtilis* MTCC441	*E. coli* MTCC2939	*E. coli* clinical	*P. aeruginosa* MTCC 424	*A. baumanii* MTCC1425	*Acinetobacter* sp. clinical	*M. smegmatis* MTCC 6
RK-1*	16.33 ±1.24	18.33±0.47	14.66±1.24	11.66±1.24	11.33±0.47	-	-	-	-
DV1D*	17.00±0.81	12.33±1.24	-	17.33±1.67	18.33±0.47	-	-	-	-
DV1H*	--	-	19.33±1.24	15.33±1.24	15.00±0.81	-	-	-	-
DV1S*	24.00 ± 0.81	23.66 ± 2.05	32.33 ± 0.47	16.66 ± 0.94	11.66 ± 0.94	-	23.66 ± 1.88	20.33 ± 0.47	21.66 ± 0.47
BR-3*	16.66±0.47	18.00±0.00	21.66±0.47	15.00±1.41	-	-	-	-	-
TRII (ii)*	11.00±0.81	13.66±0.47	9.66±1.67	9.66±0.47	-	12.66±0.94	-	-	-
JH 1*	30.33±0.94	25.00±0.81	31.00±0.81	-	-	-	-	-	-
JH 2*	19.00±1.41	16.66±0.47	29.66±0.94	11.33±0.47	-	-	-	-	
GR9a-5**	16.00 ± 1.41	13.33 ± 0.94	20.00 ± 1.24	16.00 ± 0.47	15.00 ± 0.81	14.00± 0.94	19.33 ± 0.81	15.00 ± 1.00	16.33 ± 0.94
GR4-3*	16.33±1.24	-	29.33±0.47	-	-	-	-	-	-
Rifampicin 5 µg/disc	22.33 ± 0.47	31.66 ± 1.24	17.67 ± 0.47	28.33 ± 0.47	21.00 ± 0.00	-	19.33± 0.94	17.66 ± 0.47	23.66 ± 0.94
Vancomycin (30 µg/disc)	24.00 ± 0.81	18.66 ± 0.94	21.33 ± 0.94	-	-	-	-	-	ND

* Extracted with ethyl acetate;

** Extracted with methanol; Average of triplicate ± standard deviation; ND, not determined, the diameter of the filter paper disks (6 mm) is included; (–) not active; values are mean ± standard deviation of three experiments in replicate.

**Table 5:** Phenotypic characteristics of isolates DV-1S and GR9a-5.

Characteristics	DV-1S	GR9a-5
Acid fast	-	+
Aerial mycelium	Greyish pink	Pink
Reverse	Brown	Colourless
Diffusible pigment	+, Brown	-
Melanin pigment	-	-
Sporulation	Good	-
Spore chain	Open spirals	Fragmentation
Starch hydrolysis	+	+ + +
Casein hydrolysis	-	-
Gelatin hydrolysis	-	-
Oxidase	-	+
Catalase	-	+
C- utilization		
Dextrose	+ + +	+ + +
Rhamnose	-	+ + +
D-Maltose	+ +	+ + +
L-Arabinose	-	-
L-Sucrose	-	+
L- Raffinose	-	-
Cellobiose	-	+
D-Mannose	-	+
N- Utilization		
L-Arginine	-	-
L- Valine	+ +	+ + +
L- Serine	-	+
L- Phenylalanine	+ + +	+ +
L- Threonine	+ + +	+ +
L- Methionine	+ + +	+ + +
Hydroxyproline	+ + +	+ + +
L- Histidine	+ +	+
Pottasiun nitrate	+ + +	+
Nitrate reduction	-	+ + +
Growth at temp.		
4^○^C	-	-
15- 37° C	+	+
45^○^C	-	+
Growth at NaCl (w/v)		
0-3%	+ + +	+ + +
4-6%	+ +	-
7%	+	-
Growth at pH		
4	-	+
7- 10	+	+

## DISCUSSION

Searching new antibiotics has increased worldwide because of the serious problem of antibiotic resistance among the microbes. The recent discovery of novel primary and secondary metabolites from taxonomically unique population of actinomycetes suggest that these organisms could add a new dimension to microbial natural product research. The history of new drug discovery processes shows that novel skeletons have, in the majority of cases, come from natural sources (17). On the basis of above facts new actinomycetes strains that generate active compounds have been recently isolated from novel sources including saline, ocean, mangrove forests and niche habitats such as caves, pristine forests, lakes, rivers, and other wetlands (18). Today, the emphasis is on the exploration of unusual and previously ignored ecosystems (19). Actinomycetes from several unexplored environments have been intensively studied in last few decades for novel and potent molecules (5). In this context, Uttarakhand hills (including foot hills) though less explored but possesses a great biodiversity had been selected for this study. This diversity can be explored for isolation and characterization of native actinomycetes for antimicrobial molecules. As per our knowledge this is the first exhaustive screening program done in the Uttarakhand region for isolation and screening of actinomycetes. The results of the present study revealed that the viable count of actinomycetes varied greatly with altitude. The significant differences in the viable count of actinomycetes were recorded for three different altitudes. This is in accordance with the previous reports from Sikkim, India (20). The actinomycetes isolated from these sites were belonged to the 14 different genera of actinomycetes. The total number of actinomycete genera recovered is much lower than that in similar studies performed in other geographic areas (21–23). Mainly the genus *Streptomyces* was dominant in all the soil samples; however some rare genera were also recovered in the study, but in low frequency. When considering the pH of the soil samples highest percentage of actinomycetes were recovered at alkaline while lowest from acidic soil, supporting the earlier reports (24).

The results of antibacterial activity (23.44%) in present study were different from those of other authors showing 53– 61% in Algerian soil (25). The antibacterial results were also comparable with that described by Barakate *et al*. (26) studying the activity of Moroccan soil actinomycetes and those of other authors showing 16% isolates showing antimicrobial activity in soil of Turkey (27). Highest percentages of actinomycetes were found to belong the genus *Streptomyces* (67%). This high frequency of antimicrobial activities among *Streptomyces* species has been previously observed in other soil and aquatic isolates (28–29). The results of the present study were also comparable with a previous study of actinomycetes in terrestrial soil from Doon Valley (30).

Isolate DV1S and GR9a-5 showed highest antibacterial properties against several multi-drug resistant bacterial pathogens and were identified using polyphasic approach. DV1S and GR9a-5 were found to be most closely related with *S. massasporeus* NBRC 12796^T^ and *Nocardia nova* JCM 6044^T^ respectively. These isolates can be differentiated from type strains in a number of cultural and physiological tests. Isolate DV1S produced grey aerial and brown reverse mycelium with spiral spore chains with spiny spore surface. It utilizes only glucose and arabinose and gave positive result for H_2_S production. It can tolerate a salt concentration of 7% (w/v). In contrast *S. massasporeus* produced grey to violet aerial and violet reverse mycelium with spiral spore chains with smooth spore surface and utilizes the glucose, arabinose, sucrose, inositol, manose, and fructose. It can tolerate a salt concentration of 5% (w/v) (31). Similarly, *S. indiaensis* (previously it was classified as *Streptosporangium*) produced grey aerial and brown reverse mycelium with spiral spore chain with smooth spore surface and utilized glucose, arabinose, sucrose, inositol, mannose, fructose, and rhamnose. It can tolerate a salt concentration of 5%, w/v (32). *S. hawaiiensis* produced blue to grey aerial and reverse yellow to brown mycelium with spiral spore chain with spiny spore surface. It utilized glucose, arabinose, inositol, mannose, fructose, rhamnose and raffinose (33). Hence it may be a new strain of *Streptomyces*.

Similarly, the strain GR9a-5 can be differentiated from type strains in various biochemical characteristics. *N. nova* utilized rhamnose, sucrose, inositol, L-proline, L- serine, and L- valine and whereas D- mannose, L-phenyl alanine and L- leucine were not utilized (34). In contrast strain GR9a-5 utilized rhamnose, sucrose, D-mannose, L- valine, L-serine while L- arabinose, L-raffinose, fructose, inositol, xylose, salicin, trehalose and L- arginine were not utilized. *N. jiangxiensis* utilized D- arabinose, D-cellobiose, D-fructose, glucose, inositol, D-lactose, D-maltose, D-raffinose, D-ribose, D-sorbose, D-sucrose, D-trehalose and D-xylose are used as sole carbon source. It does not degrade starch and Tween 80 (35) while, they were degraded by the strain GR9a-5. Hence, it may represent a new strain of *Nocardia*.

The results of this study strongly support the idea that the viable count of actinomycetes varied greatly with altitude. Highest viable counts of actinomycetes were recorded in valleys followed by mid hills and High hills. The actinomycetes species isolated from valleys, mid hills and high hills possess a significant capacity to produce compounds having unique antibacterial activity. The isolates DV1S and GR9a-5 were found to be most prominent in the terms of desirable activities. These isolates were found to have excellent antimicrobial potential against several drug resistant bacterial pathogens. Results obtained from this work are promising and hence merit further studies concerning purification, characterization and identification of the active secondary metabolites.
